# Baseline intact fibroblast growth factor 23 and risk of kidney disease progression in the Indian Chronic Kidney Disease cohort: a prospective multicenter study

**DOI:** 10.3389/fmed.2025.1707350

**Published:** 2026-01-05

**Authors:** Kajal Kamboj, Ashok Kumar Yadav, Aman Rastogi, Arpita Ghosh, Shubham Sharma, Love Jain, Vivek Kumar, Vivekanand Jha, Indian Chronic Kidney Disease Study Network

**Affiliations:** 1 Department of Experimental Medicine and Biotechnology, Postgraduate Institute of Medical Education and Research, Chandigarh, India; 2 The George Institute for Global Health, New Delhi, India; 3 Department of Nephrology, Postgraduate Institute of Medical Education and Research, Chandigarh, India; 4 School of Public Health, Imperial College, London, United Kingdom; 5 Prasanna School of Public Health, Manipal Academy of Higher Education, Manipal, India

**Keywords:** chronic kidney disease, CVD mortality, fibroblast growth factor 23, kidney failure, major adverse kidney events

## Abstract

**Background:**

Circulating levels of fibroblast growth factor 23 (FGF23) increase early in chronic kidney disease and are associated with a faster progression and increased mortality. However, evidence from South Asia is limited. We investigated the association between baseline intact FGF23 levels and adverse kidney outcomes in the ICKD cohort.

**Methods:**

A prospective cohort of adult participants with mild to moderate CKD enrolled at 11 Indian hospitals was included if baseline FGF-23 levels were available. Plasma iFGF-23 was measured using a two-site ELISA. The primary endpoint was major adverse kidney events (MAKE: a composite of kidney failure, ≥50% decline in eGFR, or kidney death). Secondary endpoints included individual MAKE components, all-cause mortality, and cardiovascular mortality. Cox proportional hazards models were used to evaluate the associations between iFGF23 and time-to-event outcomes.

**Results:**

A total of 602 participants were followed up for a median duration of 5.3 years. MAKE developed in 266 (49.3%) participants; 223 (41.3%) progressed to kidney failure; 211 (43.5%) reached ≥50% eGFR decline; and 66 (11.0%) died. iFGF23 was significantly associated with MAKE (SHR 1.23, 95% CI 1.02–1.47, *p* = 0.027), kidney failure (SHR 1.28, 95% CI 1.04–1.58, *p* = 0.02), and all-cause mortality (HR 1.39, 95% CI 1.05–1.83, *p* = 0.02) in unadjusted and age- and sex-adjusted Cox proportional hazards models. However, in the fully adjusted model with clinical variables, none of the associations remained statistically significant.

**Conclusion:**

In this prospective cohort of Indian CKD patients, iFGF23 levels did not provide independent prognostic information after accounting for established risk factors. Routine iFGF23 testing has limited incremental prognostic value in this setting.

## Introduction

Chronic kidney disease (CKD) is a major global public health issue. An important consequence of CKD is disordered phosphate handling and the consequent changes in mineral metabolism (1). Recognition of the central role of fibroblast growth factor 23 (FGF-23), primarily secreted by osteocytes, has been a key advance in understanding mineral metabolism abnormalities and their consequences for health in people with CKD (2, 3). Circulating FGF23 levels increase early during CKD and promote phosphaturia, which serves as an adaptive mechanism. However, it also suppresses 1-α-hydroxylase activity, reducing calcitriol (3, 4).

Observational studies and clinical trials have shown an association between raised FGF-23 and mortality, heart failure, and CKD progression in multiple cohorts (5–8). The Chronic Renal Insufficiency Cohort (CRIC) study showed FGF23 was a stronger predictor of mortality than established cardiovascular risk factors (5). Similarly, the Atherosclerosis Risk in Communities (ARIC) study found that higher baseline FGF23 levels predicted kidney failure over a 21-year follow-up period (9). However, evidence from lower-middle-income countries, including South Asia, is limited. The Indian Chronic Kidney Disease (ICKD) study (10, 11) represents the largest prospective cohort of CKD patients in LMICs and shows characteristics different from Western populations, such as distinct etiologies of CKD, a younger age at presentation, and unique socioeconomic factors. The mineral metabolism abnormalities are more severe (higher phosphate, PTH, and FGF23 levels and low calcium) in the diabetic CKD population in the CRIC cohort (12). In addition, dietary and ethnic factors can influence FGF23 levels in the CKD population (13, 14). Given these differences and established ethnic variations in mineral metabolism, validation of biomarker associations in diverse populations is crucial.

We evaluated whether baseline intact FGF-23 (iFGF-23) predicts kidney disease progression in the Indian Chronic Kidney Disease (ICKD) cohort.

## Materials and methods

The ICKD study is a multicenter, prospective cohort study recruiting patients with mild to moderate CKD from 11 large hospitals across India. Eligible participants were adults aged 18–75 years with eGFR 15–60 mL/min/1.73m^2^ or eGFR ≥60 mL/min/1.73m^2^ with proteinuria >500 mg/day. For this analysis, we included a subset of ICKD cohort participants randomly selected for FGF23 testing, who were enrolled between the study initiation and March 2020. We excluded individuals lacking iFGF23 measurement, those without follow-up, or those with missing covariates required for the adjustment models. ICKD was approved by ethics committees at participating centers, and all participants gave written informed consent.

Plasma iFGF-23 was measured at baseline using a two-site ELISA (Immutopics, Inc., San Clemente, CA, Cat. 60-6500). The assay had intra-assay precision of 4.6%, inter-assay precision of 6.5%, and sensitivity of 1 pg/mL. All samples were processed according to the manufacturer’s specifications by trained laboratory personnel who were blinded to the clinical outcomes.

The primary endpoint was major adverse kidney events (MAKE), a composite of kidney failure (initiation of dialysis or transplantation), ≥50% decline in eGFR from baseline, or death due to kidney disease. Secondary endpoints included individual MAKE components, all-cause mortality, and cardiovascular mortality. Outcome definitions were as per prespecified cohort protocols, confirmed by medical record review.

### Statistical analysis

Continuous variables are presented as mean ± standard deviation or median (25th, 75th percentiles) based on distribution. Categorical variables are presented as frequencies and percentages. iFGF23 was analyzed as a continuous variable after log transformation due to skewed distribution.

Cox proportional hazards models were used to evaluate the associations between iFGF23 and time-to-event outcomes without competing risk, and the Fine-Grey sub-distribution hazard model to evaluate the association for outcomes including competing risk. Non-kidney death was treated as a competing risk for kidney outcomes, and non-cardiovascular death was the competing risk for CV deaths. Three sequential models were constructed: unadjusted, adjusted for age and sex, and a third one additionally adjusted for hypertension, diabetes, cardiovascular disease, baseline eGFR, and urine albumin-to-creatinine ratio.

All analyses were performed using R version 4.4.2 statistical software, with two-sided *p*-values <0.05 considered significant.

## Results

The study included 602 participants, with a mean age of 47.6 ± 12.4 years, and 64% were men (Table 1). The median eGFR was 43.0 (IQR 36–55) mL/min/1.73m^2^. Hypertension was present in 507 (84%) participants, diabetes in 165 (27%), and cardiovascular disease in 78 (13%). The characteristics of the overall ICKD cohort and those included in the current study are shown in Supplementary Table S1.

**Table 1:** Baseline characteristics of participants.

Characteristics	Females (*N* = 217)	Males (*N* = 385)	Total (*N* = 602)
Demographic characteristics			
Age (years)	47.2 (11.7)	47.9 (12.7)	47.6 (12.4)
BMI (kg/m^2^)	25.5 (22.4, 29.5)	24.3 (21.5, 27.4)	24.7 (21.9, 27.7)
Waist/hip ratio	0.93 (0.87, 0.97)	0.96 (0.91, 1.02)	0.94 (0.89, 1.00)
Non-vegetarian diet	63 (29.3)	163 (42.9)	226 (38)
Clinical characteristics			
History of hypertension	175 (80.6)	332 (86.2)	507 (84.2)
History of diabetes	40 (18.4)	125 (32.5)	165 (27.4)
History of CVD	22 (10.2)	56 (14.5)	78 (13.0)
Causes of CKD			
Diabetic kidney disease	26 (12.0)	68 (17.7)	94 (15.6)
Chronic interstitial nephritis	38 (17.5)	80 (20.8)	118 (19.6)
Unknown	67 (30.9)	85 (22.1)	152 (25.2)
Glomerulonephritis	32 (14.7)	70 (18.2)	102 (17.0)
Hypertensive nephrosclerosis	12 (5.5)	16 (4.2)	28 (4.7)
Polycystic kidney disease	12 (5.5)	14 (3.6)	26 (4.3)
CAKUT	8 (3.7)	1 (0.2)	9 (1.5)
Others	22 (10.2)	51 (13.2)	73 (12.1)
Laboratory parameters			
iFGF23 (pg/mL)	125 (92, 176)	104 (74, 153)	112 (78, 163)
Hemoglobin (mg/dL)	11.2 (10.2, 12.2)	13 (11.5, 14.4)	12.2 (10.9, 13.8)
Serum creatinine (mg/dL)	1.6 (1.4, 1.9)	1.7 (1.5, 2.0)	1.7 (1.5, 2.0)
eGFR (mL/min/1.73m^2^)	38 (32, 44)	48 (39, 58)	43 (36, 55)
Serum urea (mg/dL)	50 (40, 68)	47 (37, 59)	48 (38, 62)
Serum calcium (mg/dL)	9.0 (8.7, 9.5)	9.3 (8.7, 9.6)	9.2 (8.7, 9.6)
Serum inorganic phosphorus (mg/dL)	3.9 (3.5, 4.4)	3.4 (3.1, 4.0)	3.7 (3.2, 4.2)
Serum albumin (mg/dL)	4.2 (3.9, 4.4)	4.4 (4.0, 4.7)	4.3 (4.0, 4.6)
Serum uric acid (mg/dL)	6.8 (5.5, 8.1)	7.4 (6.1, 8.5)	7.2 (5.9, 8.4)
Total cholesterol (mg/dL)	176 (150, 220)	165 (136, 197)	168 (140, 205)
Triglycerides (mg/dL)	158 (108, 216)	145 (107, 199)	150 (108, 205)
HbA1c (%)	5.55 (5.3, 6.4)	5.9 (5.4, 6.94)	5.8 (5.3, 6.8)
Urine albumin creatinine ratio (mg/g)	24.1 (11.7, 191.0)	22.2 (10.7, 212.3)	23.4 (10.7, 201.3)

Data presented as mean ± standard deviation, number (percentage) and median (25th, 75th percentile).

BMI, body mass index; DBP, diastolic blood pressure; eGFR, estimated glomerular filtration rate; FGF, fibroblast growth factor HbA1c, glycosylated hemoglobin; SBP, systolic blood pressure.

The median baseline iFGF23 level was 112 (IQR 78–163) pg/mL. Women had significantly higher iFGF23 levels than men (125 vs. 104 pg/mL; *p* < 0.001). iFGF23 levels were inversely correlated with eGFR, with the highest levels observed in participants with eGFR <30 mL/min/1.73m^2^ (median 157 pg/mL) compared to those with eGFR ≥60 mL/min/1.73m^2^ (median 96 pg/mL, *p* < 0.001) (Supplementary Table S2).

**Figure 1:**
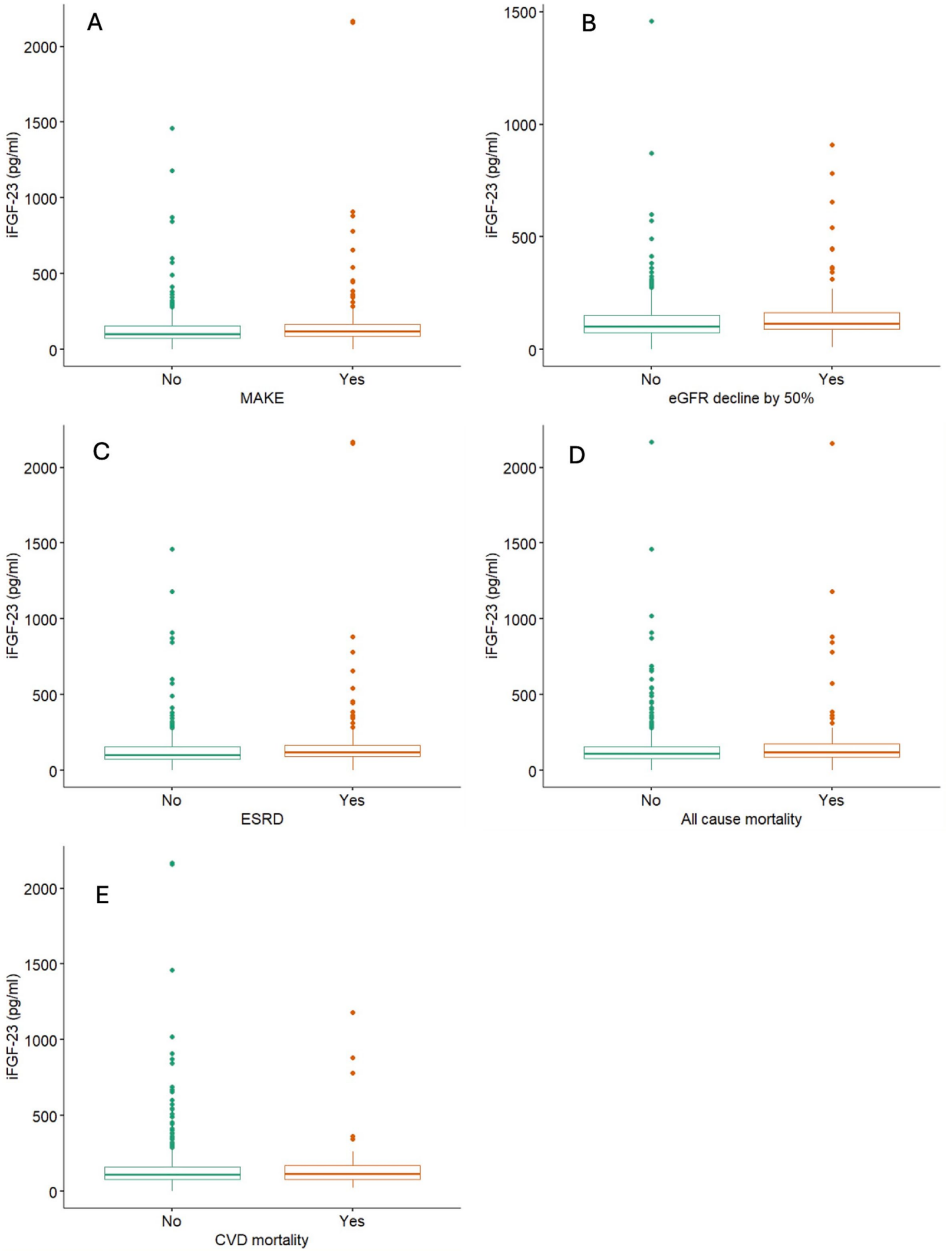
Levels of iFGF23 based on outcome events. **(A)** MAKE, **(B)** ≥50% eGFR decline, **(C)** ESRD (kidney failure), **(D)** all-cause mortality, and **(E)** CVD mortality in ICKD cohort. MAKE, major adverse kidney events; ERKD, end stage renal disease; eGFR, estimated glomerular filtration rate; CVD, cardiovascular disease.

During a follow-up of 5.3 ± 2.4 years, 266 (49.3%) experienced MAKE; 223 (41.3%) progressed to kidney failure; 211 (43.5%) reached ≥50% eGFR decline; and 66 (11.0%) died. Participants who developed MAKE had higher baseline iFGF23 levels than those who did not (median 121 vs. 103 pg/mL; *p* < 0.001). Similar patterns were observed for kidney failure (121 vs. 103 pg/mL, *p* < 0.001) and ≥50% eGFR decline (116 vs. 104 pg/mL, *p* = 0.002). No significant differences were observed for all-cause or cardiovascular mortality (Figure 1).

In unadjusted analysis, higher iFGF23 was significantly associated with increased risk of MAKE (SHR 1.57, 95% CI 1.23–1.99, *p* = 0.017), kidney failure (SHR 1.30, 95% CI 1.06–1.60, *p* = 0.013), and all-cause mortality (HR 1.43, 95% CI 1.10–1.87, *p* = 0.008) but not with ≥50% eGFR decline or cardiovascular mortality.

**Table 2:** Association of intact FGF-23 with outcomes.

Outcomes	Model 1 sub-hazard ratio (95% CI)	Model 2 sub-hazard ratio (95% CI)	Model 3 sub-hazard ratio (95% CI)
MAKE	1.24 (1.04; 1.49) [0.017]	1.23 (1.02; 1.47) [0.027]	1.13 (0.94, 1.36) [0.19]
50% eGFR decline	1.15 (0.98; 1.35) [0.09]	1.14 (0.97; 1.34) [0.12]	1.10 (0.93, 1.30) [0.25]
Kidney failure	1.30 (1.06; 1.6) [0.013]	1.28 (1.04; 1.58) [0.02]	1.11 (0.90, 1.37) [0.31]
All-cause mortality^*^	1.43 (1.1; 1.87) [0.008]	1.39 (1.05; 1.83) [0.02]	1.26 (0.96, 1.66) [0.09]
CVD mortality	1.49 (0.95; 2.34) [0.08]	1.53 (0.97; 2.4) [0.07]	1.47 (0.93, 2.34) [0.10]

Model 1 was unadjusted. Model 2 was adjusted for age and gender. Model 3 was adjusted for variables in Model 2 + hypertension, diabetes, CVD, baseline eGFR, and urine albumin-to-creatinine ratio.

^*^Hazard ratio is reported.

CVD, cardiovascular disease; MAKE, major adverse kidney events; eGFR, estimated glomerular filtration.

Figures in square brackets represent *p*-value.

After adjustment for age and sex (Model 2), the associations remained significant for MAKE (SHR 1.23, 95% CI 1.02–1.47, *p* = 0.027), kidney failure (SHR 1.28, 95% CI 1.04–1.58, *p* = 0.02), and all-cause mortality (SHR 1.39, 95% CI 1.05–1.83, *p* = 0.02). However, in the fully adjusted model (Model 3), none of the associations remained statistically significant. The sub-hazard ratios were substantially attenuated: MAKE (SHR 1.13, 95% CI 0.94–1.36, *p* = 0.19), kidney failure (SHR 1.11, 95% CI 0.90–1.37, *p* = 0.31), and all-cause mortality (HR 1.47, 95% CI 0.93–2.34, *p* = 0.09) (Table 2).

### Sensitivity analysis

When iFGF23 was dichotomized at the median (112 pg/mL), similar patterns were observed. Participants with above-median iFGF23 had increased risks of MAKE (SHR 1.57, 95% CI 1.23–1.99, *p* < 0.001), kidney failure (SHR 1.62, 95% CI 1.24–2.10, *p* < 0.001), and all-cause mortality (HR 1.73, 95% CI 1.19–2.51, *p* = 0.004) in unadjusted models, and the associations remained significant after full adjustment for MAKE: SHR 1.44, 95%CI;1.12; 1.85, *p* = 0.005 and kidney failure: SHR 1.36, 95% CI 1.03–1.79, *p* = 0.032 but not for all-cause mortality: HR 1.41, 95% CI 0.96–2.08, *p* = 0.08 (Supplementary Table S3).

## Discussion

In this analysis of the ICKD cohort, we found that iFGF23 was associated with adverse kidney outcomes in univariate and minimally adjusted models, but these associations were not independent of established CKD risk factors after full adjustment. This contrasts with findings from several Western cohorts, such as CRIC (5, 8) and ARIC (9), where FGF23 has been identified as an independent predictor of outcomes even after comprehensive adjustment. The MMKD study also found that FGF23 is an independent predictor of CKD progression (6). The HOST study reported an independent association of FGF23 with all-cause mortality, cardiovascular events, and initiation of chronic dialysis in the early advanced stage of CKD (15). However, the CARE FOR HOMe study revealed a more nuanced association, with FGF23 being linked to future decompensated heart failure but not to incident atherosclerotic events in stage 2–4 CKD (16).

Possible reasons for these differences include differences in population characteristics. Our cohort had a lower median age than typical Western CKD cohorts. Younger patients may have different underlying pathophysiology and risk profiles that modify the prognostic utility of FGF23. The differences in CKD etiology (a higher proportion of CKDu and CIN and a lower proportion of DN) (11) may indicate distinct mineral metabolism patterns compared to diabetic kidney disease, which predominates in Western cohorts. Finally, ethnic variations in mineral metabolism, dietary patterns, and genetic polymorphisms affecting FGF23 metabolism may influence its prognostic value.

From a clinical standpoint, our findings suggest that iFGF23 measurement may not provide additional prognostic information beyond standard clinical variables in Indian patients with CKD. This has implications for resource allocation and biomarker implementation in LMICs, where cost-effectiveness is a critical consideration. However, FGF23 may still have therapeutic implications, and interventional studies targeting FGF23 or its downstream effects could provide insights into causality and potential treatment targets, even if the biomarker itself has limited independent prognostic value.

Our study has several strengths—it is a large multicenter study with a prospective design, standardized outcome adjudication, and comprehensive covariate assessment. Limitations include a single baseline iFGF23 measurement, which precludes assessment of longitudinal changes, and a lack of data on medications that might influence FGF23 levels. Additionally, we did not assess the effects of phosphate or parathyroid hormone on FGF23, which may confound or modify the association between iFGF23 and outcome. Differences in the diet, baseline history of CVD, and causes of CKD between current cohort and entire ICKD cohort may also limit the generalizability of study findings.

To conclude, in this prospective cohort of Indian CKD patients, iFGF23 levels were associated with adverse kidney outcomes in univariate analysis but did not provide independent prognostic information beyond established clinical variables. Our study suggests that the clinical utility of iFGF23 as a prognostic biomarker may vary across populations and clinical contexts, emphasizing the importance of validating biomarkers across diverse populations before recommending widespread clinical adoption.

## Data Availability

The original contributions presented in the study are included in the article/Supplementary material, further inquiries can be directed to the corresponding author.

## Group member of Indian Chronic Kidney Disease Network

Ajay Jaryal, Department of Nephrology, Indira Gandhi Medical College, Shimla, India; Asheesh Kumar Kapil, Department of Nephrology, Indira Gandhi Medical College, Shimla, India; Gopesh Modi, Samarpan Kidney Institute and Research Centre, Bhopal, India; Kaushik Bhattacharjee, Department of Nephrology, Institute of Post Graduate Medical Education & Research, Kolkata, India; N. Gopalakrishnan, Rajiv Gandhi Government General Hospital, Chennai, India; Narayan Prasad, Department of Nephrology, Sanjay Gandhi Postgraduate Institute of Medical Science, Lucknow, India; Manisha Sahay, Department of Nephrology, Osmania Medical College, Osmania General Hospital, Hyderabad, India; Sanjay Vikrant, Department of Nephrology, Indira Gandhi Medical College, Shimla, India; Santosh Varughese, Department of Nephrology, Christian Medical College, Vellore, India; Sishir Gang, Department of Nephrology Muljibhai Patel Urological Hospital, Nadiad, India; Sreejith Parameswaran, Department of Nephrology, Jawaharlal Institute of Postgraduate Medical Education & Research, Pondicherry, India; R. Sakthirajan, Rajiv Gandhi Government General Hospital, Chennai, India; Seema Baid Agarwal, Department of Nephrology and Transplant Center, Sahlgrenska University Hospital, University of Gothenburg, Sweden.
