# Supplementary material for: Baseline intact fibroblast growth factor 23 and risk of kidney disease progression in the Indian Chronic Kidney Disease cohort: a prospective multicenter study
Source: Front Med (Lausanne). 2026 Jan 5;12:1707350. doi: 10.3389/fmed.2025.1707350 (PMC12812529; doi:10.3389/fmed.2025.1707350)
Supplement: Supplementary file 1 [file Data_Sheet_1.pdf]

**Supplementary Material**

**Supplementary Table 1: Comparison of baseline data of ICKD cohort and subjects analysed for this study**

| Characteristics | ICKD cohort (N=4318 ) | Current analysis (N=602 ) |
|---|---|---|
| **Demographic characteristics** | | |
| **Sex (Male/Female)** | 2891 (70)/1427 (30) | 385 (64)/217 (36) |
| **Age (years)** | 50.2 (11.8) | 47.6 (12.4) |
| **BMI (kg/m2)** | 24.3 (21.6, 27.3) | 24.7 (21.9, 27.8) |
| **Waist/hip ratio** | 0.95 (0.92, 0.98) | 0.94 (0.89, 1.00) |
| **Non-vegetarian diet** | 2,756 (65.7) | 226 (38) |
| **Clinical Characteristics** | | |
| **History of Hypertension** | 3705 (85.8) | 507 (84.2) |
| **History of Diabetes** | 1,499 (34.7) | 165 (27.4) |
| **History of CVD** | 918 (21.6) | 78 (13.0) |
| **Causes of CKD** | | |
| **Diabetic kidney disease** | 1,065 (25.2) | 94 (15.6) |
| **Chronic interstitial nephritis** | 964 (22.8) | 118 (19.6) |
| **Unknown** | 802 (18.9) | 152 (25.2) |
| **Glomerulonephritis** | 626 (14.9) | 102 (17.0) |
| **Hypertensive Nephrosclerosis** | 336 (7.9) | 28 (4.7) |
| **Polycystic Kidney disease** | 149 (3.5) | 26 (4.3) |
| **KACUT** | 39 (0.9) | 9 (1.5) |
| **Others** | 243(5.9) | 73 (12.1) |
| **Laboratory parameters** | | |
| **Haemoglobin (mg/dl)** | 11.8 (10.5, 13.2) | 12.2 (10.9, 13.8) |

| | | |
|---|---|---|
| **Serum creatinine (mg/dl)** | 1.7 (1.5, 2.0) | 1.7 (1.5, 2.0) |
| **eGFR (ml/min/1.73m$^2$)** | 43 (36, 53) | 43 (36, 55) |
| **Serum urea (mg/dl)** | 45 (34, 56) | 48 (37, 62) |
| **Serum calcium (mg/dl)** | 9.0 (8.5, 9.5) | 9.2 (8.7, 9.6) |
| **Serum inorganic phosphorus (mg/dl)** | 3.9 (3.3, 4.4) | 3.7 (3.2, 4.2) |
| **Serum albumin (mg/dl)** | 4.0 (3.5, 4.3) | 4.3 (4.0, 4.6) |
| **Serum uric acid (mg/dl)** | 6.4 (5.3, 7.6) | 7.2 (5.9, 8.4) |
| **Total cholesterol (mg/dl)** | 166 (134, 200) | 168 (140, 205) |
| **Triglycerides (mg/dl)** | 140 (110, 177) | 150 (108, 205) |
| **HbA1c (%)** | 5.70 (5.10, 6.80) | 5.8 (5.3, 6.8) |
| **Urine albumin creatinine ratio (mg/g)** | 53.9 (10.7, 399.6) | 23.4 (10.7, 201.3) |

Data represented as mean±stadandard deviation, median (25th, 75th percentile) and number (percentage)

BMI; body mass index, CVD: cardiovascular disease, CKD: chronic kidney disease, eGFR; estimated glomerular filtration rate, uACR; urine albumin to creatinine ratio

**Supplementary Table 2:** levels of iFGF23 in various subgroups

| Variables | Subgroups | iFGF23(pg/mL) N = 602 | P-value |
|---|---|---|---|
| **Gender** | Female (n=217) | 125 (92, 175) | <0.001 |
| | Male (n= 385) | 104 (74, 153) | |
| **Age (years)** | ≥50 (n=297) | 114 (82, 162) | 0.53 |
| | <50 (n=305) | 109 (76, 166) | |
| **Hypertension** | No (n=95) | 103 (74, 137) | 0.10 |
| | Yes (n=507) | 113 (79,168) | |
| **Diabetes** | No (n=437) | 109 (78,158) | 0.71 |
| | Yes (n=165) | 123 (81,169) | |
| **eGFR (ml/min/1.73m$^2$)** | <30 (n=52) | 157 (113, 231) | <0.001* |
| | 30-44.9 (n=279) | 121 (87, 175) | |
| | >45-59.9 (n=159) | 99 (75, 149) | |
| | >60 (n=110) | 96 (72, 125) | |

Data presented as **Median (25$^{th}$, 75$^{th}$** percentile**)**, P value obtained using Mann Whitney T test, * The Kruskal-Wallis test

**Supplementary Table 3**. Association of intact FGF-23 with Outcomes in CKD patients with respect to the Median Value of iFGF23

| Outcome | Model 1 | Model 2 | Model 3 |
|---|---|---|---|
| MAKE | 1.57 (1.23;1.99) [p<0.001] | 1.60 (1.25;2.05) [p <0.001] | 1.44 (1.12; 1.85) [p =0.005] |
| Kidney failure | 1.62 (1.24; 2.10) [p <0.001] | 1.65 (1.26;2.17) [p <0.001] | 1.36 (1.03; 1.79) [p =0.031] |
| ≥50% decline in eGFR | 1.42 (1.08;1.86) [p =0.011] | 1.45 (1.10;1.91) [p =0.008] | 1.37 (1.03; 1.82) [p =0.032] |
| CVD death | 1.27 (0.69;2.33) [p =0.44] | 1.24 (0.68;2.28) [p =0.48] | 1.17 (0.65; 2.10) [p =0.60] |
| All cause death | 1.73 (1.19;2.51) [p =0.004] | 1.57 (1.07;2.30) [p =0.02] | 1.41 (0.96; 2.08) [p =0.08] |

Model 1 was unadjusted. Model 2 was adjusted for age and gender. Model 3 was adjusted for variables in model 2 + hypertension, diabetes, CVD, baseline eGFR, and urine albumin-to-creatinine ratio.

*Hazard ratio is reported.

MAKE, major adverse kidney events; CVD: cardiovascular disease, eGFR, estimated glomerular filtration.
